# Faecal blood loss with aspirin, nonsteroidal anti-inflammatory drugs and cyclo-oxygenase-2 selective inhibitors: systematic review of randomized trials using autologous chromium-labelled erythrocytes

**DOI:** 10.1186/ar2355

**Published:** 2008-01-17

**Authors:** R Andrew Moore, Sheena Derry, Henry J McQuay

**Affiliations:** 1 Pain Research, Nuffield Department of Anaesthetics, University of Oxford, Oxford Radcliffe Hospitals, The Churchill, Headington, Oxford, OX3 7LJ, UK

## Abstract

**Introduction:**

Faecal blood loss has been measured using autologous erythrocytes labelled with radioactive chromium for several decades, using generally similar methods. We conducted a systematic review of studies employing this technology to determine the degree of blood loss associated with use of aspirin, nonsteroidal anti-inflammatory drugs (NSAIDs) and cyclo-oxygenase-2 selective inhibitors (coxibs).

**Methods:**

A systematic search of PubMed and the Cochrane Library (to December 2006) was conducted to identify randomized trials in which treatment with aspirin, NSAIDs, or coxibs was continued for at least 7 days, and with at least 7 days of washout for crossover trials. Rates of faecal blood loss associated with these agents were determined in the randomized trials identified. Comparators were placebo, active, or no treatment. Outcomes of interest were mean daily faecal blood loss, and the number or proportion of individuals recording faecal blood above 5 ml/day and above 10 ml/day.

**Results:**

Forty-five reports of 47 trials were included, including 1,162 individuals, mostly healthy volunteers and predominantly young men. Only 136 patients (as opposed to healthy volunteers; 12%) were included, and these were mostly older people with an arthritic condition. Most NSAIDs and low-dose (325 mg) aspirin resulted in a small average increase in faecal blood loss of 1 to 2 ml/day from about 0.5 ml/day at baseline. Aspirin at full anti-inflammatory doses resulted in much higher average levels of blood loss of about 5 ml/day. Some individuals lost much more blood than average, at least for some of the time, with 5% of those taking NSAIDs having daily blood loss of 5 ml or more and 1% having daily blood loss of 10 ml or more; rates of daily blood loss of 5 ml/day or 10 ml/day were 31% and 10%, respectively, for aspirin at daily doses of 1,800 mg or greater.

**Conclusion:**

At baseline, or with placebo, faecal blood loss is measured at 1 ml/day or below. With low-dose aspirin and some NSAIDs, average values may be two to four times this, and anti-inflammatory doses of aspirin result in much higher average losses. A small proportion of individuals respond to aspirin or NSAIDs with much higher faecal blood loss of above 5 ml/day or 10 ml/day. There are significant limitations regarding the quality and validity of reporting of these studies, such as limited size and inclusion of inappropriate participants. The potential for blood loss and consequent anaemia requires more study.

## Introduction

Nonsteroidal anti-inflammatory drugs (NSAIDs) are effective analgesics and anti-inflammatory drug therapy is an important pharmacological approach to treating various forms of pain, chronic musculoskeletal pain in particular. NSAIDs have a number of known adverse effects. NSAIDs (and aspirin) are associated with upper gastrointestinal injury [1], acute renal failure [2,3] and congestive heart failure [4,5]. Less well documented adverse events include associations with increased fracture rates [6] and lower gastrointestinal injury [7-9]. The latter includes bleeding [10-16] and permeability changes [17-19]. Cyclo-oxygenase-2 selective inhibitors (coxibs) are differentiated from traditional NSAIDs by lower rates of upper and lower gastrointestinal harm, and possibly by lack of effect on bone.

The gastrointestinal outcomes most often reported in modern, large, randomized trials and observational studies are upper gastrointestinal bleeding [20-22] or hospital admission for upper gastrointestinal bleeding [23-26]. Both outcomes represent a serious and significant clinical event that is probably at one extreme of a spectrum of blood loss. Much less is known about lower gastrointestinal bleeding and low-level chronic blood loss. Measurements of blood loss to the entire bowel demonstrate large differences between individuals, with some individuals losing significant amounts of blood on a daily basis, up to 50 ml or more [27,28].

The clinical significance of low-level blood loss is unclear. Morris and colleagues [29] found small bowel lesions in 10 out of 15 patients with both rheumatoid arthritis and anaemia. In randomized trials anaemia was less common when patients were treated with celecoxib rather than NSAIDs [30], and there was lower rate of bowel injury with coxibs [14].

Various methods have been used to measure blood loss from the whole bowel [18,31-33]. The use of radioactively labelled autologous erythrocytes with concomitant measurement of radioactivity in blood and faeces has been longest used. The method involves stool collection for a number of days after injection of ^51^Cr-erythrocytes. Methodological problems, notably those involving patients with long transit times [34], collection of all stool samples, avoidance of interfering behaviours and suitable methods for measuring radioactivity in blood and stool, were identified early on. Many randomized trials have been conducted over a number of decades using essentially similar methods. Typically, they compared the effects of aspirin, NSAID, or coxib on mean daily faecal blood loss, with comparators of placebo or aspirin. We chose to examine these trials systematically, both for effects on mean daily blood loss across groups and to identify individuals with greater levels of blood loss that might be connected with anaemia.

## Materials and methods

Quality of Reporting of Meta-analyses guidelines were followed where appropriate [35]. PubMed and the Cochrane Library were searched to identify randomized trials using the autologous radioactive chromium method to measure faecal blood loss with aspirin, NSAIDs, or coxibs. The date of the last search was December 2006. A series of free text terms was used, using combinations of words in title and abstract, including faecal (or fecal) blood loss, occult blood loss, chromium, erythrocyte*, aspirin, N-acetylsalicylic acid, NSAID, nonsteroidal anti-inflammatory drug, cyclooxygenase-2 inhibitor, as well as the individual names of common drugs, including ibuprofen, diclofenac, naproxen, indomethacin, ketoprofen, rofecoxib, celecoxib, etoricoxib, valdecoxib and lumiracoxib. Electronic searches were supplemented by exploration of bibliographies of all papers obtained, and reviews of gastrointestinal damage caused by aspirin or NSAIDs. Trials identified as possibly relevant from title or abstract were obtained in full paper version.

Trials were included if they were randomized; if they used chromium-labelled autologous erythrocytes to measure faecal blood loss with collections in controlled conditions; if they had at least one placebo arm in the trial; if they involved administration of any dose of aspirin, NSAID, or coxib for at least 7 days; and if they were parallel group or had a crossover design with at least 7 days of washout between therapies.

Information from each trial was abstracted into a table. Relevant information concerned randomization, blinding, and withdrawal and dropouts was collected to assess reporting quality using a commonly used 5-point scoring system [36]. Methodological items noted were as follows: whether a history was taken to exclude possible pre-existing causes of intestinal bleeding; whether participants had a regular bowel habit; dependence of inclusion on either a negative faecal occult blood test or a measured low faecal blood loss on baseline screening; whether the study was conducted on an inpatient basis in controlled conditions; and how the method of calculating faecal blood loss was reported.

Other information noted included information on study design, participants (age, sex, volunteer or patient), treatments (including dose), duration, baseline faecal blood loss and daily faecal blood loss at the longest time period. As well as noting mean or median blood loss, we sought information about the number of participants with faecal blood loss of greater than 5 ml/day or greater than 10 ml/day.

It was recognized that trials would be heterogeneous in relation to active therapies used, including drug dose. The intention was to calculate weighted mean faecal blood loss using results from individual treatment arms reporting mean or median results, with weighting by number of participants in the study group. It was expected that dispersion information (standard deviation or standard error of the mean) would be irregularly reported. In addition, the proportion of participants with faecal blood loss above 5 ml/day or above 10 ml/day was calculated.

It was expected that for aspirin the dose range might be large, with studies using full-dose aspirin (arbitrarily set at ≥1,800 mg/day) or low-dose aspirin (arbitrarily set at ≤325 mg/day). Results within these two dose ranges would be calculated separately. For NSAIDs and coxibs, no extreme variation in dose was envisaged, but the analysis plan allowed for exclusion of very high or very low doses if these were outside the usual daily dose range (for example, ibuprofen 800 to 2,400 mg/day, diclofenac 50 to 150 mg/day and naproxen 500 to 1,000 mg/day). The analysis plan also allowed for comparison of results from healthy volunteers and patients. No other sensitivity analyses were planned, and no statistical testing was done.

## Results

**Figure 1:**
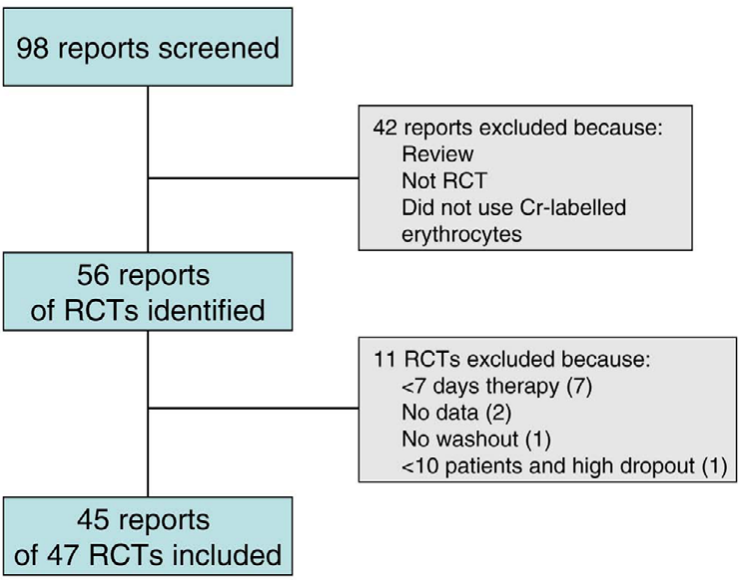
Flow diagram for study selection. RCT, randomized controlled trial.

Figure 1 is a flow chart showing the choice of studies for inclusion. Of papers examined in detail, 43 did not involve chromium-labelled erythrocytes, were not randomized trials, or were reviews, and six appeared to be possible randomized trials but they did not include a drug under investigation. Ten randomized trials did involve both chromium-labelled autologous erythrocytes and a drug under review, but their duration was less than 7 days (seven trials), had no information for the first phase only of a crossover trial (two), had no washout between treatments in a crossover study (one), or included only eight participants in different trials, with data available on four for any treatment (one).

There remained 45 reports of 47 randomized trials (Additional file 1 includes details of the trials, including trial design; nature of participants, and their sex, age and any illness; faecal blood loss results; and references). Two reports [37,38] included extractable information on two randomized trials, and one [28] had additional information and analysis of two randomized trials [39,40]. The trials had been published over 40 years (Table 1); 26 were described as double blind, and 25 had quality scores of 3 or more out of 5 (Table 1). Thirty-four trials reported therapy periods of 1 week (22 trials) or 2 weeks (12 trials), published mainly before the mid-1980s; 13 trials reported therapy periods of 3 weeks (two trials) or 4 weeks or longer (10 trials), published mainly since the mid-1980s.

**Table 1:** Trial quality by decade of publication

Decade	Quality score					Total
		
	1	2	3	4	5	
1960s				2		2
1970s		5		3	1	9
1980s	1	11	5	7	2	26
1990s		5		3		8
2000s			1		1	2
Total	1	21	6	15	4	47

A number of methodological criteria might reflect on the validity of a trial. For example, only 36 out of 47 trials indicated that patients had been screened to exclude a pre-existing history that might contribute to increased faecal blood loss, such as a prior history of gastrointestinal disease or surgery; oral, nasal, or rectal bleeding (including bleeding gums on brushing them); or haemorrhoids. Almost all trials excluded current or recent use of drugs that are likely to interfere with measurements; typically, these were analgesics or low-dose aspirin, and recent prior use of aspirin, NSAIDs, antacids, histamine antagonists, or proton pump inhibitors. Behavioural issues leading to exclusion were excessive alcohol use, excessive use of caffeinated beverages, or peculiarities of diet. Only 12 trials used a negative faecal occult blood test as an entry criterion, and only five used a baseline low daily faecal blood loss to exclude participants. Regular bowel habit was an entry criterion in 11 trials, whereas only five trials used controlled conditions (inpatient or dormitory accommodation). Detailed reporting of the method of measuring faecal and blood radioactivity was uncommon, and although most reported at least a reference four trials had neither details nor reference. Only two trials [39,40] reported adequately on all of these points.

Almost all studies involved a baseline, pretreatment faecal blood loss measurement over a period of days, and elevated baseline faecal blood loss was also a reason for exclusion (and occasionally investigation). The total number of individuals investigated was 1,162. Most were healthy volunteers, predominantly young men; only 136 (12%) were patients, usually older with an arthritic condition.

**Figure 2:**
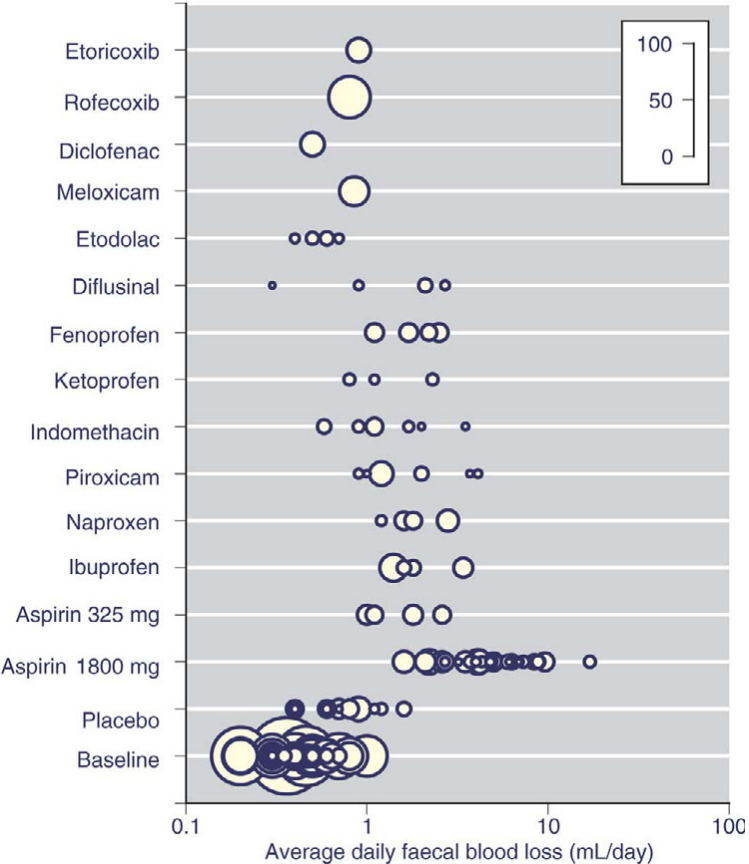
Mean daily faecal blood loss in individual treatment arms. Daily faecal blood loss is shown on a logarithmic scale for aspirin, cyclo-oxygenase-2 selective inhibitors (coxibs) and nonsteroidal anti-inflammatory drugs (NSAIDs) with more than 20 participants. The size of the symbol is proportional to the number of individuals (inset scale).

Table 2 shows the weighted mean daily faecal blood loss for baseline measurements before treatment, on placebo and for each treatment. For aspirin, data are divided according to dose, with trials examining low-dose aspirin (all 325 mg/day) or full-dose aspirin (≥1,800 mg/day). Daily mean faecal blood loss for individual study arms is shown in Figure 2 for baseline, placebo, coxibs and NSAIDs with at least two trials and 25 patients.

**Table 2:** Weighted mean daily faecal blood loss by therapy

Treatment/drug	Studies or study arms (*n*)	Participants (*n*)	Weighted mean faecal blood loss (ml/day)
Initial blood loss at start of trial	38	950	0.46
Placebo arm of treatment	14	172	0.76
Aspirin (>1,800 mg/day)	29	361	5.01
Aspirin (325 mg/day)	4	64	1.61
Ibuprofen	4	66	2.03
Fenoprofen	4	62	1.86
Piroxicam	6	60	1.89
Naproxen	4	59	1.98
Indomethacin	6	58	1.39
Etodolac	4	39	0.55
Rofecoxib	1	37	0.80
Diflusinal	4	33	1.68
Ketoprofen	3	28	1.42
Meloxicam	1	26	0.85
Etoricoxib	1	21	0.90
Diclofenac	1	21	0.50
Azapropazone	2	20	1.00
Suoprofen	1	20	1.80
Nefopam	1	19	0.60
Tiaprofen	2	17	0.51
Zomepirac	2	16	6.40
Oxindanac	1	16	1.30
Sulindac	2	15	0.75
Lornoxicam	1	15	0.80
Bromfenac	1	12	2.10
Fluproquazone	1	12	2.80
Nabumetone	1	10	1.60
Oxaprozin	1	10	2.30
Isoxicam	1	8	1.00
Ticlotil	1	8	2.10
Tenoxicam	1	6	1.20
Flurbiprofen	1	6	1.30

Note that all low dose aspirin was 325 mg/day.

Most information on mean daily faecal blood loss was available for baseline, the initial period before randomization and the start of the trial proper. This was available in 38 trial arms and 950 participants. Baseline mean daily faecal blood loss was below 1 ml/day in all but one study [41], in which it was 1.0 ml/day; the weighted mean was 0.46 ml/day. A somewhat higher mean daily faecal blood loss of 0.76 ml/day was measured with placebo in 172 participants (Table 2).

Mean daily faecal blood loss was available for low-dose and full-dose aspirin, 25 different NSAIDs, and rofecoxib and etoricoxib. There was information on 361 participants for aspirin at doses greater than 1,800 mg/day, but for other aspirin doses, NSAIDs and coxibs there was information on fewer than 100 participants, and mostly fewer than 50 participants. For 16 NSAIDs information available was on 20 individuals or fewer (Table 2).

**Figure 3:**
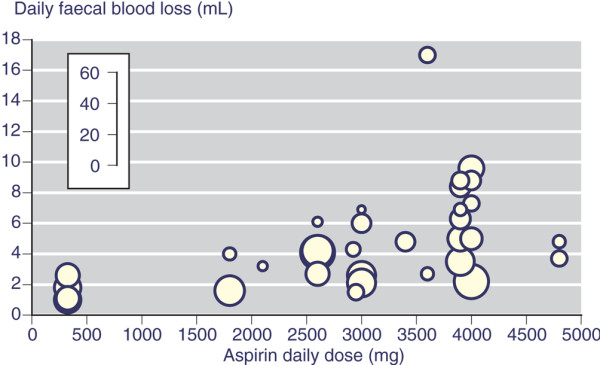
Average daily faecal blood loss in individual trials of aspirin according to daily aspirin dose. The size of the symbol is proportional to the number of individuals (inset scale).

For aspirin there appeared to be a dose-response relation (Figure 3), with maximum weighted mean values of up to 4 ml/day at 2,000 mg/day, 6 ml/day at 3,000 mg/day, and 10 ml/day at 4,000 mg/day. The most commonly used NSAIDs had mean daily faecal blood loss values of between 1 and 2 ml/day (ibuprofen and naproxen 2.0 mL/day), apart from diclofenac, meloxicam and etodolac, with values of about 0.5 to 0.9 ml/day (Table 2 and Figure 2). Rofecoxib and etoricoxib also had low mean daily faecal blood loss of 0.8 and 0.9 ml/day.

At baseline there was no obvious difference between healthy young volunteers (0.44 ml/day, 835 individuals) and patients (0.56 ml/day, 103 individuals). With aspirin at doses above 1,800 mg/day, the mean daily faecal blood loss was about twice as high with volunteers (5.8 ml/day, 249 individuals) as with patients (3.2 ml/day, 112 individuals).

A number of the studies either gave information on individual patients or provided suitable information to identify whether any participants individually had daily faecal blood loss above 5 or 10 ml/day. For instance, a range of individual mean values was often provided. Such information permitted determination that all patients had faecal blood loss of under than 5 and 10 ml/day if the upper value of the range was below 5 ml/day, but if the top of the range was above 10 mL/day then this identified only one such individual; others who may have had higher values could not be identified by this method, which therefore provides a minimum estimate.

The estimated number and percentage of patients who individually had higher blood loss is shown in Table 3. Blood loss greater than 5 ml/day did not occur with placebo in any participant. For aspirin at 1,800 mg/day or more, 31% of participants had blood loss of 5 ml/day or greater, and 10% had loss of 10 ml/day or greater. Lower rates of 5% and 1%, respectively, were found for all NSAIDs combined.

**Table 3:** Number and percentage of individuals with higher daily faecal blood loss

Therapy	Individuals (*n*)	Faecal blood loss (*n *[%])	
		
		>5 ml/day	>10 ml/day
Placebo	149	0 (0%)	0 (0%)
Aspirin ≥1,800 mg/day	309	97 (31%)	31 (10.0%)
Any NSAID	469	24 (5.1%)	4 (0.9%)

NSAID, nonsteroidal anti-inflammatory drug.

## Discussion

Systematic reviews have several utilities. They can shed new light on a topic or resolve a difference of opinion, but sometimes they can indicate only what is in the literature, without extensive analysis. For any analysis to make sense, it must be based on information that is of sufficient quality to avoid bias, it must be valid (at least within a reasonable definition for a topic), and the volume of data analyzed must be of sufficient size to prevent conclusions from being wafted about on the winds of chance.

Systematic reviews can only attempt to make sense of the information presented. In the case of the present review, the number of trials of sufficient quality limited, increasing the likelihood of bias. We have defined validity, *inter alia*, by duration of therapy at a minimum of 1 week and washouts of 1 week in crossover studies. Analysis is limited by clinical factors, design and reporting heterogeneity. There were only 1,162 participants in 47 trials (average 25/trial, split between several groups in the parallel group trials). There were different levels of reporting quality, both crossover and parallel designs, duration periods of 1 to 4 weeks, and 30 different treatments (including placebo, and some at different doses); the bulk of studies were conducted in young healthy men, but some were conducted in young healthy women, and a minority (12%) evaluated older patients. Other variables include whether studies maintained participants in a controlled environment to collect stool samples reliably, and the mechanics of measuring radioactivity in stool samples. In the face of so many variables, our judgement was that no statistical approach could possibly be justified. Although we would like to know how any of these variables might have affected the results, we cannot see how such an analysis may be achieved and so we resorted to a descriptive analysis.

Given the clear limitations of quality and validity in these individually small trials, even a descriptive analysis can only be undertaken with circumspection. Comment is, however, still required on the results that are available.

In these randomized trials, conducted over five decades, some (but not all) of the drugs investigated resulted in a small average increase in faecal blood loss of 1 to 2 ml/day. Aspirin appeared to be associated with increased faecal blood loss in a dose-dependent manner; full anti-inflammatory doses (>1,800 mg/day) resulted in much higher average levels of blood loss of about 5 ml/day, although even 325 mg/day resulted in daily faecal blood loss equivalent to that with standard dose NSAIDs. For aspirin and NSAIDs, some individuals lost much more blood on average, with 5% of those taking NSAIDs having daily blood loss of 5 ml or more and 1% having daily blood loss of 10 ml or more. Individual daily blood loss above 50 ml was reported in some healthy young men with ibuprofen [28], and with aspirin over as few as 5 days [32], and individual rather high blood loss was reported sporadically.

These headline results must be qualified in several ways. Most importantly, almost 90% of individuals investigated were healthy, most were young (age <40 years) and most were men. It is not known whether these results can be extrapolated to older populations, often with comorbid conditions, using aspirin or NSAIDs. We found no conclusive evidence that baseline faecal blood loss, or faecal blood loss with aspirin at doses above 1,800 mg/day, was higher in patients than in healthy volunteers.

Other than obvious differences between trials in terms of drugs and doses tested, the main difference between trials was in the duration of therapy. We set a minimum limit of 7 days of treatment, in part to limit under-estimation of faecal blood loss caused by slow intestinal transit time [34]. More recent studies have tended to include longer treatment duration and older studies tended to involve shorter treatment durations, but there was an insufficient number of common therapies to allow a sensible comparison to be conducted, especially because the number of patients in each treatment arm was small. There was the added complication of crossover trials, with disparate washout periods, although a minimum of 7 days was imposed as an inclusion criterion.

Additional limitations arose from the apparent differences between trials in participant inclusion. Although most trials screened participants for a history of potential for increased gastrointestinal damage, or drugs that might interfere or confound measurements, fewer than one-third excluded participants with higher blood loss by means of faecal occult blood screening or measurement. Moreover, only one trial in 10 collected stool samples in controlled conditions from inpatients, and full description of the methods used to measure stool or blood radioactivity was uncommon. All of these features indicated that a cautious descriptive analysis of these data was all that was possible.

Although frank bleeding from the upper and lower gastrointestinal tracts is associated with high levels of anaemia, including very low haemoglobin levels (<100 g/l) [42], there is no direct evidence linking anaemia with low-level blood loss into the bowel, although aspirin, NSAIDs and perhaps coxibs are known to be associated with varying frequencies of frank gastrointestinal bleeding. There is circumstantial evidence that NSAIDs are linked both to increased faecal blood loss and to increased anaemia [30]. For low-dose aspirin, which produced faecal blood loss similar to that of NSAIDs in these trials, a limited body of literature has examined only small numbers, with one study [43] suggesting an association between low-dose aspirin use and anaemia and another one [44] finding no association. A small comparative study of misoprostol and no treatment in 21 patients with small bowel enteropathy and iron deficiency anaemia showed a rise of 15 g/l in haemoglobin with misoprostol, as compared with no change without treatment [45].

It is interesting that individual participants could have bleeding rates that were much higher than the average, more frequently with full-dose aspirin than with NSAIDs. It is tempting to consider that individuals with daily faecal blood loss of more than 5 ml or 10 ml might be more likely to develop anaemia, especially because some individuals had daily blood loss of 50 ml or more (for example, in the study conducted by Bowne and coworkers [28]). Caution is needed, however, because in most cases the information we obtained indicates that high blood loss was identified over just a few days, and we have no good evidence about the longer term. The exception [28], a re-analysis of ibuprofen used in healthy male volunteers, indicated that very high blood loss may occur infrequently and intermittently. Longer studies and larger numbers would be needed to demonstrate the cause and effect between daily faecal blood loss and anaemia.

Anaemia is common in older people living in the community; 13% of a Canadian population of 17,000 had anaemia using the World Health Organization definition (<120 g/l for women and <130 g/l for men), and 4% had haemoglobin levels below 110 g/l [46]. Anaemia is also common (prevalence about 50%) in some rheumatological conditions [47]. Of 72,000 older patients admitted to hospital with myocardial infarction, 43% satisfied a World Health Organization definition of anaemia [48]. Anaemia in older people is associated with greater mortality in association with heart failure [49,50], angina [51] and myocardial infarction, especially with compromised renal function [52]. Anaemic older patients in hospital are less likely to resume activities of daily living [53] and anaemia is associated with poor cognition [54].

Where a cause for anaemia can be found and treated, quality of life improves. This is the case with blood transfusion in older people, or when haemoglobin levels rise upon treatment with erythropoietin in cancer [55] or renal disease. There are no reliable studies demonstrating improvements in hard clinical outcomes or mortality.

## Conclusion

At baseline, or with placebo, faecal blood loss is generally measured at 1 ml/day or below. With low-dose aspirin and some NSAIDs, average values in studies may be two to four times this, and with anti-inflammatory doses of aspirin there is consistent evidence for much higher average losses. A small proportion of individuals respond to aspirin or NSAIDs with much higher faecal blood loss of above 5 ml/day or above 10 ml/day. Blood loss of 5 ml/day would represent almost 2 l if continued over a year, a not insubstantial loss, which might be a consequence of NSAID use in up to one in 20 users. Similar concerns apply to low-dose aspirin. There are significant limitations regarding the quality and validity of reporting of these studies, such as limited size and inclusion of inappropriate participants. The potential for blood loss and consequent anaemia requires more study.

## Abbreviations

coxib = cyclo-oxygenase-2 selective inhibitor; NSAID = nonsteroidal anti-inflammatory drug.

## Competing interests

RAM and HJM have received consulting and/or lecture fees from pharmaceutical companies and other organizations. The authors have received research support from charities and government sources at various times. No author has any direct stock holding in any pharmaceutical company.

## Authors' contributions

RAM, HJM and SD were involved with the original concept and planning of the study. RAM performed searches, and led on data extraction, analysis and preparing the manuscript. SD helped with data extraction, analysis and writing. HJM helped with writing and advice. All authors read and approved the final manuscript.

## Supplementary Material

Additional file 1 A PDF file which contains information on each included study, with reference, quality score, design, treatments, main results, and comments. Click here for file
