# Supplementary material for: Faecal blood loss with aspirin, nonsteroidal anti-inflammatory drugs and cyclo-oxygenase-2 selective inhibitors: systematic review of randomized trials using autologous chromium-labelled erythrocytes
Source: Arthritis Res Ther. 2008 Jan 17;10(1):R7. doi: 10.1186/ar2355 (PMC2374474; doi:10.1186/ar2355)
Supplement: Additional file 1 — A PDF file which contains information on each included study, with reference, quality score, design, treatments, main results, and comments. [file ar2355-S1.pdf]

Additional file 1: Details of the randomised trials included in the review

Study<br>Quality score	Design<br>Treatments	Results	Comments
Bowen et al. Clin Gastroenterol Hepatol 2005 3: 1075-1082<br>R = 2<br>DB = 2<br>W = 1<br>QS = 5	Post-hoc analysis of two RCTs with ibuprofen as control. Cr51-labelled RBC to measure faecal erythrocyte excretion in controlled conditions over 28 days<br>Healthy male volunteers aged 18-60 years<br>Placebo (n=37)<br>Ibuprofen 2,400 mg daily (n=31)	Placebo run in average faecal blood loss was 0.36 mL/day.<br>No subject on placebo had >2 mL/day, with daily mean of 0.7 mL/day (±0.4 SD).<br>On ibuprofen the mean daily blood loss was 2.6 mL/day (±3.2 SD).<br>28-day blood loss averaged 20 mL for placebo and 71 mL for ibuprofen, but was between 100 and 300 mL in 5 patients	Note that patients here are second analysis of two RCTs below (Hunt, 2000, 2003).<br>Individual daily blood loss on ibuprofen with micro bleeding episodes, episodic in nature. All had >2 mL/day average loss. 27/31 on ibuprofen had from 2-7 episodes of >3 mL/day, 5 subjects 4 episodes >2 and <3mL/day. Nine subjects had maximum blood loss >10 mL/day, with about 70 mL/day in two<br>5/31 lost >3.5 mL/day on average over 28 days<br>2/31 lost >6 mL/day on average over 28 days
Hunt et al. Aliment Pharmacol Ther 2003 17: 201-210<br>R = 2<br>DB = 1<br>W = 1<br>QS = 3	Cr51-labelled RBC to measure faecal erythrocyte excretion in controlled conditions over 28 days<br>Healthy male volunteers aged 18-33 years<br>Placebo (n=21)<br>Ibuprofen 2,400 mg daily (n=17)<br>Etoricoxib 120 mg daily (n=21)	Average daily blood loss end week 4:<br>Placebo 0.9 mL/day<br>Ibuprofen 2,400 mg daily 3.4 mL/day<br>Etoricoxib 120 mg daily 0.9 mL/day	Mean values taken from figure. No dispersion data available
Hunt et al. Am J Med 2000 109: 201-206<br>R = 2<br>DB = 2<br>W = 1<br>QS = 5	Cr51-labelled RBC to measure faecal erythrocyte excretion in controlled conditions over 28 days<br>Healthy male volunteers aged 18-60 years (mean 23 years)<br>Placebo (n=17)<br>Rofecoxib 25 mg daily (n=18)<br>Rofecoxib 50 mg daily (n=19)<br>Ibuprofen 2400 mg daily (n=13)	Average daily blood loss at baseline was about 0.4 mL/day<br>Over weeks 2-4:<br>Placebo 0.7 mL/day<br>Rofecoxib 0.8 mL/day<br>Rofecoxib 0.8 mL/day<br>Ibuprofen 1.8 mL/day	Mean values taken from table No dispersion data available

Van Hecken et al. Drug Metab Drug Interact 1998 14: 193-205<br>R = 1<br>DB = 0<br>W = 1<br>QS = 2	Cr51-labelled RBC to measure faecal erythrocyte excretion in controlled conditions over 4-7 days of 7 days treatment in two crossover groups randomised for addition of clopidrogel<br>Healthy male volunteers mean 22 years<br>Both groups (n=15) received placebo followed by naproxen 500 mg daily	Average daily blood loss at baseline was about 0.3 mL/day<br>Over last 3 or 4 days of study period:<br>Group 1<br>Placebo 0.4 (±0.4 SD) mL/day<br>Naproxen 1.1 (±0.6) mL/day<br>Group 2<br>Placebo 0.3 (±0.2 SD) mL/day<br>Naproxen 1.9 (±1.5) mL/day<br>Maximum daily blood loss with naproxen was 2.4 mL/day in group 1 and 5.8 mL/day in group 2	Another treatment period also examined the effects of clopidrogel. Without clopidrogel mean daily blood loss was 1.8 mL/day (maximum 5.6 mL/day).<br>With clopidrogel mean daily blood loss was 6.8 mL/day (maximum 28 mL/day).
Scharf et al. Aust NZ J Med 1998 28: 436-439<br>R = 1<br>DB = 0<br>W = 1<br>QS = 2	Cr51-labelled RBC to measure faecal erythrocyte excretion in controlled conditions over 2 weeks in a crossover study<br>Patients with osteoarthritis (mean age 69 years - range 61-83 years) 19 male, 4 female<br>No treatment (n=23)<br>Diclofenac 100 mg daily (n=21)<br>Naproxen 750 mg daily (n=19)<br>Piroxicam 20 mg daily (n=21)	Average daily blood loss over 3 days at end of treatment<br>No treatment 0.3 (±0.1 SEM) mL/day<br>Diclofenac 100 mg daily 0.5 (±0.2) mL/day<br>Naproxen 750 mg daily 2.8 (±2.2) mL/day<br>Piroxicam 20 mg daily 1.2 (±0.6) mL/day	Two upper GI bleeding events (diclofenac, piroxicam) and one lower GI bleeding event (diclofenac) occasioned withdrawals
Patoia et al. J Rheumatol 1996 35 (suppl 1): 61-67<br>R = 1<br>DB = 2<br>W = 1<br>QS = 4	Cr51-labelled RBC to measure faecal erythrocyte excretion in controlled conditions over 28 days<br>Healthy male volunteers aged 19-34 years<br>Placebo (n=13)<br>Meloxican 7.5 mg daily (n=13)<br>Meloxican 15 mg daily (n=13)<br>Piroxicam 20 mg daily (n=12)	Basline values averaged 0.2 mL/day, all 0.5 mL/day or less<br>Average daily blood loss days 1-28:<br>Placebo 0.6 (±0.4 SD) mL/day<br>Meloxican 7.5 mg daily 0.8 (±0.5) mL/day<br>Meloxican 15 mg daily 0.9 (±0.7) mL/day<br>Piroxicam 20 mg daily 2.0 (±4.1) mL/day<br>Maximum average daily blood loss was 1.9, 2.1, 2.7 and 15 mL daily	Blood loss tended to be similar for each of four weeks of treatment<br>6/12 patients taking piroxicam were withdrawn due to clinically relevant mucosal damage on endoscopy

Savon et al. Am J Gastroenterol 1995 90: 581-585<br>**(trial 1)**<br>R = 1<br>DB = 0<br>W = 1<br>QS = 2	Cr51-labelled RBC to measure faecal erythrocyte excretion in controlled conditions over 7 days in crossover study<br>Healthy volunteers aged 19-36 years (17 male, 3 female)<br>Placebo<br>Plain aspirin 325 mg daily<br>Enteric coated aspirin 325 mg daily	17 completers; baseline blood loss 0.5 (±0.1 SEM) mL/day<br>Average daily blood loss based on 72-hour stool collection at end of treatment<br>Plain aspirin 325 mg daily 1.8 (±0.4) mL/day<br>Enteric coated aspirin 325 mg daily 1.0 (±0.1) mL/day	
Savon et al. Am J Gastroenterol 1995 90: 581-585<br>**(trial 2)**<br>R = 1<br>DB = 0<br>W = 1<br>QS = 2	Cr51-labelled RBC to measure faecal erythrocyte excretion in controlled conditions for minimum of 63 days<br>Healthy volunteers aged 21-56 years (38 male, 2 female)<br>Plain aspirin 325 mg daily (n=15)<br>Enteric coated aspirin 325 mg daily (n=15)	30 completers<br>Average daily blood loss based on 72-hour stool collection at end of treatment<br>Plain aspirin 325 mg daily 2.6 (±0.7) mL/day<br>Enteric coated aspirin 325 mg daily 1.1 (±0.3) mL/day	No difference between shorter and longer term aspirin consumption for faecal blood loss
Cohen. Clin Ther 1995 17: 1110-1116<br>R = 1<br>DB = 2<br>W = 1<br>QS = 4	Cr51-labelled RBC to measure faecal erythrocyte excretion in controlled conditions over 21 days<br>Healthy male volunteers aged 18-45 years (mean age 30 years)<br>Placebo (n=12)<br>Aspirin 3,900 mg daily (n=13)<br>Bromfenac 300 mg daily (n=12)	Basline values averaged 0.5 mL/day<br>Average daily blood loss days over last 3 days:<br>Placebo 0.4 (±0.2 SD) mL/day<br>Aspirin 3,900 mg daily 8.4 (±4.2) mL/day<br>Bromfenac 300 mg daily 2.1 (±1.1) mL/day<br>Maximum individual daily faecal blood loss about 3 mL/day with bromfenac, 19 mL/day with aspirin	9/13 on aspirin had daily blood loss >5 mL/day<br>3/13 on aspirin had daily nl;ood loss >10 mL/day

Güntert et al. Psychopharmacology 1992 106: S40-S42 (Duplicate)<br>Güntert et al. Drug Metab Drug Interact 1991 10: 307-322<br>R = 1<br>DB = 0<br>W = 0<br>QS = 2	Cr51-labelled RBC to measure faecal erythrocyte excretion in controlled conditions over 7 days. Randomisation was to moclobemide or placebo, with ibuprofen 1,800 mg introduced on day 8-14 in both groups (12 per group)<br>Healthy male volunteers aged 19-40 years (n=24)	Baseline faecal blood loss 0.4 mL/day<br>Average daily blood loss over last 4 days of ibuprofen administration:<br>Ibuprofen + placebo 1.5 (±1.0 SD) mL/day<br>Ibuprofen + moclobemide 1.3 (±0.6) mL/day	Moclobemide made no difference to faecal blood loss or ibuprofen kinetics. Randomisation and blinding was not by ibuprofen use, so inclusion of this trial is questionable
Warrington et al. Postgraduate Medical J 1990 66: 622-626<br>R = 1<br>DB = 2<br>W = 1<br>QS = 4	Cr51-labelled RBC to measure faecal erythrocyte excretion in controlled conditions over 28 days<br>Healthy male volunteers aged 21-35 years<br>Placebo (n=15)<br>Lornoxicam 8 mg daily (n=15)<br>Indomethacin 150 mg daily (n=15)	Median daily blood loss days over last 7 days:<br>Placebo 0.6 mL/day (maximum 13 mL/day)<br>Lornoxicam 0.8 mL/day (maximum 2.7 mL/day)<br>Indomethacin 1.1 mL/day (maximum 2.1 mL/day)	No dispersion given<br>1/15 on placebo had daily blood loss >10 mL/day<br>Individual patient data available
Patoia et al. Eur J Clin Pharmacol 1989 36: 599-604<br>R = 1<br>DB = 2<br>W = 1<br>QS = 4	Cr51-labelled RBC to measure faecal erythrocyte excretion in controlled conditions over 28 days<br>Healthy young volunteers (12 male, 9 female)<br>Placebo (n=7)<br>Piroxicam 20 mg daily (n=7)<br>Piroxicam beta-cyclodextrin 20 mg daily (n=7)	Baseline faecal blood loss 0.3 mL/day<br>Mean daily blood loss days over last 7 days:<br>Placebo 0.4 (±0.2 SEM) mL/day<br>Piroxicam 4.1 (±2.6) mL/day<br>Piroxicam beta-cyclodextrin 1.2 (±0.4) mL/day	Steady increase with piroxicam over 28 days, but 4/7 withdrew over the period with adverse events. Large increases in faecal blood loss in second and third weeks also. In first week mean blood loss 0.5, 1.6 and 1.0 mL/day respectively<br>At least one patinet on piroxicam must have had daily blood loss >5 mL/day

Lynch et al. Aust NZ Med J 1989 19: 89-96<br>R = 1<br>DB = 0<br>W = 0<br>QS = 1	Cr51-labelled RBC to measure faecal erythrocyte excretion in controlled conditions over 28 days (14 days with treatment)<br>Patients with osteoarthritis aged 18 to 75 years (14 male, 26 female)<br>Buffered aspirin 4,000 mg daily (N=22)<br>Enteric coasted aspirin 3,900 mg daily (n=18)	Baseline faecal blood loss 0.7 mL/day (maximum 1.4 mL/day)<br>Average daily blood loss over last 4 days of aspirin administration:<br>Buffered aspirin 2.2 mL/day (maximum 6.6 mL/day)<br>Enteric coated 3.5 mL/day (maximum 11 mL/day)	During final week:<br>1/18 enteric coated aspirin had blood loss >10 mL/day<br>1/18 buffered aspirin had blood loss >5 mL/day<br>Individual patient data available in form of scattergram of additional blood loss
Lussier et al. J Clin Pharmacol 1989 29: 225-229<br>R = 1<br>DB = 2<br>W = 1<br>QS = 4	Cr51-labelled RBC to measure faecal erythrocyte excretion in controlled conditions over 28 days (21 days with treatment)<br>Healthy male volunteers aged 18-32 years (mean 23 years)<br>Placebo (n=10)<br>Aspirin 3,600 mg daily (n=10)<br>Nabumetone 2,000 mg daily (n=10)	Baseline faecal blood loss 0.3 mL/day<br>Average daily blood loss over last 7 days of treatment:<br>Placebo 1.2 (±2.2 ?SD) mL/day<br>Aspirin 17 (±2.2) mL/day<br>Nabumetone 1.6 (±2.2) mL/day	Note that dispersion values are not stated as SD or SEM, and that idential values for each treatment over several weeks and different means suggests the figures given may be mistaken<br>Steady increase in daily blood loss with aspirin over three weeks<br>Assumed that with mean blood loss 17 mL/day, at least 5/10 were over 10, and 7/10 over 5 mL/day
Aabakken et al. Scand J Gastroenterol 1989 24: 1007-1013<br>R = 1<br>DB = 2<br>W = 1<br>QS = 4	Cr51-labelled RBC to measure faecal erythrocyte excretion in controlled conditions over 7 days in crossover study<br>Healthy male volunteers aged 20-52 years (mean 24 years)<br>Naproxen 750 mg daily (n=16)<br>Oxindanac 600 mg daily (n=16)	Baseline faecal blood loss 0.5 mL/day<br>Average daily blood loss over 7 days of treatment:<br>Naproxen 1.6 (maximum 3.5) mL/day<br>Oxindanac 1.3 (maximum 1.9) mL/day	

Bird et al. Curr Med Res Ther 1988 11: 4-9<br>R = 1<br>DB = 2<br>W = 1<br>QS = 4	Cr51-labelled RBC to measure faecal erythrocyte excretion in controlled conditions over 14 days<br>Healthy male volunteers aged 35-67 years<br>Indomethacin Continus 150 mg daily (n=6)<br>Indomethacin R 150 mg daily (n=6)	Baseline faecal blood loss 0.6 mL/day<br>Average daily blood loss over last week of treatment:<br>Indomethacin Continus 2.0 (±0.9 SD) mL/day<br>Indomethacin R 3.5 (±3.6) mL/day	At end of therapy, 1/12 had daily blood loss >5 mL/day<br>Mean values after one week were similar, but mainly due to individuals with high blood loss
Warrington et al. Drugs 1988 35 (Suppl 1): 90-94<br>R = 1<br>DB = 0<br>W = 1<br>QS = 2	Cr51-labelled RBC to measure faecal erythrocyte excretion in controlled conditions over 28 days<br>Healthy male volunteers aged 18-34 years<br>Tiaprofic acid tablets 600 mg daily (n=7)<br>Tiaprofic acid capsules 600 mg daily (n=10)<br>Indomethacin 75 mg daily (n=10)	Baseline faecal blood loss 0.2 mL/day<br>Average daily blood loss over fourth week of treatment:<br>Tiaprofic acid tablets 0.7 mL/day<br>Tiaprofic acid capsules 0.4 mL/day<br>Indomethacin 0.9 mL/day	Mean values during second week of treatment were similar<br>All had maximum blood loss of below 5 mL/day
Malchow-Møller & Ranløv. Scand J Gastroenterol 1987 22: 550-552<br>R = 1<br>DB = 0<br>W = 1<br>QS = 2	Cr51-labelled RBC to measure faecal erythrocyte excretion in controlled conditions over 7 days in a crossover study.<br>Randomisation was to sucralfate or placebo, with aspirin 4,000 mg daily<br>Healthy male volunteers aged 20-45 years (n=16)	Baseline faecal blood loss 0.4 mL/day<br>Average daily blood loss over last 3 days:<br>Aspirin + placebo 9.6 (±1.8 SEM) mL/day<br>Aspirin + Sucralfate 7.2 (±1.6) mL/day	Individual mean daily blood loss of >10 mL/day in 6/16 on aspirin + placebo alone (maximum 25 mL/day), and 4/16 on aspirin + sucralfate<br>Individual mean daily blood loss of >5 mL/day in 10/16 on aspirin + placebo alone, and 8/16 on aspirin + sucralfate
Ryan et al. Clin Pharm Ther 1987 42: 28-32<br>R = 1<br>DB = 0<br>W = 1<br>QS = 2	Cr51-labelled RBC to measure faecal erythrocyte excretion in controlled conditions over 14 days<br>Healthy male volunteers aged 21-50 years (mean 28 years)<br>Fenoprofic acid plain 600 mg daily (n=16)<br>Fenoprofic acid enteric coated 600 mg daily (n=16)	Baseline faecal blood loss 0.2 mL/day<br>Average daily blood loss over second week of treatment:<br>Fenoprofic acid plain 1.7 (±0.9 SD) mL/day<br>Fenoprofic acid enteric coated 1.1 (±1.1) mL/day	Values at one week similar to those at two weeks<br>No patient had blood loss greater than 5 mL/day<br>Individual patient data available

Jallad et al. Am J Med Sci 1986 292: 272-276<br>R = 1<br>DB = 0<br>W = 1<br>QS = 2	Cr51-labelled RBC to measure faecal erythrocyte excretion in controlled conditions over 28 days treatment<br>Men with osteoarthritis aged 28 to 70 years (mean 57 years)<br>Etodolac 600 mg daily (n=8)<br>Etodolac 1000 mg daily (n=8)<br>Piroxicam 20 mg daily (n=6)	Baseline faecal blood loss 0.4-1.2 mL/day between groups<br>Average daily blood loss over last 4 days of treatment:<br>Etodolac 600 mg 0.7 (±0.4 SD) mL/day<br>Etodolac 1000 mg 0.4 (±0.2) mL/day<br>Piroxicam 20 mg 3.7 (±3.2) mL/day	2/6 on piroxicam had mean daily blood loss >5 mL
Bird et al. Curr Med Res Ther 1985 9: 524-528<br>R = 1<br>DB = 2<br>W = 1<br>QS = 4	Cr51-labelled RBC to measure faecal erythrocyte excretion in controlled conditions over 28 days<br>Healthy male volunteers aged over 40 years (mean 52 years)<br>Tenoxicam 20 mg daily (n=6)<br>Piroxicam 20 mg daily (n=6)	Baseline faecal blood loss 0.35 mL/day<br>Average daily blood loss over last week of treatment:<br>Tenoxicam 1.2 mL/day<br>Piroxicam 1.0 mL/day	Individual patient data available<br><br>No patient had blood loss of more than 5 mL/day
Hooper et al. Clin Pharm Ther 1985 38: 533-537<br>R = 1<br>DB = 0<br>W = 1<br>QS = 2	Cr51-labelled RBC to measure faecal erythrocyte excretion in controlled conditions over 28 days<br>Healthy male volunteers aged 18-40 years (mean 24 years)<br>Isoxicam 200 mg daily (n=8)<br>Piroxicam 20 mg daily (n=8)	Baseline faecal blood loss 0.3 mL/day<br>Average daily blood loss over last week of treatment:<br>Isoxicam 1.0 mL/day (±0.2 SD) mL/day<br>Piroxicam 0.9 mL/day (±0.2) mL/day	Values in weeks 2-4 similar<br>Data from two patients with higher levels of faecal blood loss (maximum 3.9 and 14 mL/day) omitted from piroxicam, and one (4.4 mL/day) from isoxicam. Clear spikes in blood loss in these patients<br>Individual patient data available
Salom et al. J Clin Pharmacol 1984 24: 240-246<br>R = 1<br>DB = 0<br>W = 1<br>QS = 2	Cr51-labelled RBC to measure faecal erythrocyte excretion in controlled conditions over 14 days.<br>Healthy male volunteers aged 18-47 years<br>Etodolac 800 mg daily (n=11)<br>Etodolac 1,200 mg daily (n=12)<br>Ibuprofen 2,400 mg daily (n=12)<br>Indomethacin 200 mg daily (n=9)<br>Naproxen 700 mg daily (n=9)	Baseline faecal blood loss 0.3-0.5 mL/day between groups<br>Average daily blood loss over last 3 days of treatment:<br>Etodolac 0.5 mL/day<br>Etodolac 0.6 mL/day<br>Ibuprofen 1.6 mL/day<br>Indomethacin 1.7 mL/day<br>Naproxen 1.2 mL/day	No dispersion given

Bird et al. J Clin Pharmacol 1984 24: 240-246<br>R = 1<br>DB = 2<br>W = 1<br>QS = 4	Cr51-labelled RBC to measure faecal erythrocyte excretion in controlled conditions over two 14-day crossover periods<br>Healthy volunteers aged over 35 years (mean age 50 years), 11 male, 1 female<br>Ro 21-5521 250 mg daily<br>Placebo	Baseline faecal blood loss 0.5 mL/day<br>Average daily blood loss over last 4 days of treatment:<br>Ro 21-5521 2.9 mL/day<br>Placebo 0.5 mL/day	No dispersion given
Robbins et al. Clin Ther 1984 6: 461-466<br>R = 1<br>DB = 0<br>W = 1<br>QS = 2	Cr51-labelled RBC to measure faecal erythrocyte excretion in controlled conditions over 7 days.<br>Healthy male volunteers aged 18-35 years<br>Uncoated aspirin 2,925 mg daily (n=9)<br>Enteric coated aspirin 2,950 mg daily (n=10)	Baseline faecal blood loss 0.3 mL/day<br>Average daily blood loss over last 4 days of treatment:<br>Uncoated aspirin 4.3 (±1.7 SD) mL/day<br>Enteric coated aspirin 1.5 (±0.6) mL/day	3/9 with uncoated aspirin had blood loss >5 mL/day<br>Individual patient data available
Rider. Pharmacotherapy 1983 3 (Suppl 1): 61S-64S<br>R = 2<br>DB = 2<br>W = 1<br>QS = 5	Cr51-labelled RBC to measure faecal erythrocyte excretion in controlled conditions over 14 days.<br>Healthy male volunteers<br>Diflusinal 1,000 mg daily (n=12)<br>Aspirin 4,000 mg daily (n=12)<br>Placebo	Baseline faecal blood loss 1.0 mL/day<br>Average daily blood loss over last 7 days of treatment:<br>Diflusinal 2.1 mL/day<br>Aspirin 8.8 mL/day<br>Placebo 1.6 mL/day	Does not say allocation was at random, but excellent description of blinding. Assumed to be acceptable
Ranløv et al. Scand J Rheumatol 1983 12: 280-284<br>R = 1<br>DB = 0<br>W = 1<br>QS = 2	Cr51-labelled RBC to measure faecal erythrocyte excretion in controlled conditions over 7 days.<br>Healthy male student volunteers<br>Aspirin 4,000 mg daily (n=10)<br>Ketoprofen 200 mg daily (n=10)<br>Ketoprofen SR capsule 200 mg daily (n=8)<br>Ketoprofen SR tablet 200 mg daily (n=10)	Baseline faecal blood loss <1.0 mL/day in all patients, mean 0.3 mL/day<br>Average daily blood loss over last 7 days of treatment:<br>Aspirin 7.3 mL/day<br>Ketoprofen 2.3 mL/day<br>Ketoprofen SR capsule 1.1 mL/day<br>Ketoprofen SR tablet 0.8 mL/day	8/10 with aspirin had blood loss >4 mL/day, 2 >25 mL/day<br>Individual patient data available, but not accurate values

Arnold & Berger. Pharmacology 1983 27 (suppl 1): 14-22<br>R = 1<br>DB = 1<br>W = 1<br>QS = 3	Cr51-labelled RBC to measure faecal erythrocyte excretion in controlled conditions over two 7-day crossover periods<br>Healthy male volunteers aged 23-57 years (mean age38 years, n=20)<br>Aspirin 2,600 mg daily<br>Suprofen 800 mg daily	Baseline faecal blood loss 0.4 mL/day<br>Average daily blood loss over last 4 days of treatment:<br>Aspirin 4.2 mL/day<br>Suprofen 1.8 mL/day	No dispersion given<br>Individual patient data may be available from a graph<br>Aspirin 11/20 blood loss >5 mL/day; 2/20 >10 mL/day<br>Suoprofen 2/20 blood loss >5 mL/day
Green et al. Pharmacotherapy 1983 3: 65S-69S (duplicate)<br>Green et al. Clin Pharm Ther 1981 30: 378-384<br>R = 1<br>DB = 1<br>W = 1<br>QS = 3	Cr51-labelled RBC to measure faecal erythrocyte excretion in controlled conditions over 7 days.<br>Healthy male and female volunteers aged 21-40 years<br>Placebo (n=8)<br>Aspirin 2,600 mg daily (n=6)<br>Diflusinal 500 mg daily (n=8)<br>Diflusinal 1000 mg daily (n=8)	Baseline faecal blood loss mean 0.5 mL/day<br>Average daily blood loss over last 4 days of treatment:<br>Placebo 0.6 mL/day<br>Aspirin 6.1 mL/day<br>Diflusinal 0.9 mL/day<br>Diflusinal 2.7 mL/day	No accurate dispersion given
Guercolini et al. Clinical Trials Journal 1983 20: 53-58<br>R = 1<br>DB = 2<br>W = 1<br>QS = 4	Cr51-labelled RBC to measure faecal erythrocyte excretion in controlled conditions over two 8-day crossover periods<br>Healthy youing volunteers (n=12), 10 male, 2 female<br>Indomethacin 150 mg daily<br>Glucamethicin 420 mg daily	Baseline faecal blood loss mean 0.48 mL/day<br>Average daily blood loss over 1 day in the middle of treatment:<br>Indomethacin 0.58 mL/day	Individual patient data available<br>No patient had blood loss >5 mL/day
Bird et al. Curr Med Res Op 1983 8: 412-416<br>R = 1<br>DB = 0<br>W = 1<br>QS = 2	Cr51-labelled RBC to measure faecal erythrocyte excretion in controlled conditions over two 14-day crossover periods<br>Healthy volunteers aged 34-59, 6 male, 2 female<br>Tilcotil 40 mg daily<br>Aspirin 3,600 mg daily	Baseline faecal blood loss 0.3 mL/day<br>Average daily blood loss over last 4 days of treatment:<br>Tilcotil 2.1 mL/day<br>Aspirin 2.7 mL/day	No dispersion given<br>Aspirin 1/8 blood loss >5 mL/day

Dirksen et al. Scand J Rheumatol 1982 11: 129-132<br>R = 1<br>DB = 0<br>W = 1<br>QS = 2	Cr51-labelled RBC to measure faecal erythrocyte excretion in controlled conditions over two 7-day crossover periods<br>Male outpatients (RA, AS), age 32-73 years (n=18)<br>Aspirin time release 3,000 mg daily<br>Aspirin ph release 3,000 mg daily	Baseline faecal blood loss 0.5 mL/day<br>Median daily blood loss over last 5 days of treatment:<br>Aspirin time release 2.6 mL/day<br>Aspirin ph release 2.1 mL/day	No accurate dispersion given<br>One patient (AS) had average daily blood loss of up to 77 mL/day and had low Hb level
Lussier et al. J Clin Pharmacol 1982 22: 173-178<br>R = 2<br>DB = 2<br>W = 1<br>QS = 5	Cr51-labelled RBC to measure faecal erythrocyte excretion in controlled conditions over 14 days.<br>Healthy male student volunteers aged 21-40 years<br>Placebo (n=8)<br>Aspirin 3,900 mg daily (n=11)<br>Oxaprozin 1,200 mg daily (n=10)	Baseline faecal blood loss mean 0.6 mL/day<br>Average daily blood loss over last 3 days of treatment:<br>Placebo 1.1 mL/day<br>Aspirin 8.8 mL/day<br>Oxaprozin 2.3 mL/day	No dispersion given
Gillberg et al. Scand J Rheumatol 1981 10: 342-346<br>R = 1<br>DB = 0<br>W = 1<br>QS = 2	Cr51-labelled RBC to measure faecal erythrocyte excretion in controlled conditions over two 7-day crossover periods<br>Healthy male students, age 18-31 years (n=12)<br>Aspirin 3,000 mg daily<br>Fluproquazone 300 mg daily	Baseline faecal blood loss 0.5 mL/day<br>Median daily blood loss over last 5 days of treatment:<br>Aspirin 6.0 mL/day<br>Fluproquazone 2.8 mL/day	No accurate dispersion given<br>7/12 on aspirin had daily blood loss >5 mL/day, and 2/12 ≥10 mL/day<br>4/12 on fluproquazone had daily blood loss >5 mL/day<br>Individual patient data available

Johnson. J Clin Pharmacol 1980 20: 401-405 (two trials)<br>R = 1<br>DB = 1<br>W = 1<br>QS = 3	Cr51-labelled RBC to measure faecal erythrocyte excretion in controlled conditions over 7 days.<br>Healthy male and female volunteers (17 men, 15 women)<br>Trial 1:<br>Aspirin 3,900 mg daily (n=8)<br>Zomepirac 300 mg daily (n=8)<br>Trial 2:<br>Aspirin 4,800 mg daily (n=8)<br>Zomepirac 600 mg daily (n=8)	Baseline faecal blood loss under 1.1 mL/day (average 0.8 mL/day)<br>Average daily blood loss over last 3 days of treatment:<br>Trial 1:<br>Aspirin 6.9 mL/day<br>Zomepirac 3.3 mL/day<br>Trial 2:<br>Aspirin 4.8 mL/day<br>Zomepirac 9.5 mL/day	No dispersion given<br>8/16 on aspirin had daily blood loss >5 mL/day, and 2/16 ≥10 mL/day<br>4/16 on zomepirac had daily blood loss >5 mL/day, and 3/16 ≥10 mL/day<br>Individual patient data available
Chernish et al. Arth Rheum 1979 22: 376-383<br>R = 1<br>DB = 2<br>W = 1<br>QS = 4	Cr51-labelled RBC to measure faecal erythrocyte excretion in controlled conditions over two 7-day crossover periods (placebo between active treatments)<br>Healthy male Volunteers age 23-60 years, mean 44 years (n=16)<br>Aspirin 3,900 mg daily<br>Fenoprofen 2,400 mg daily<br>Placebo	Baseline faecal blood loss not measured<br>Average daily blood loss over last 4 days of treatment:<br>Aspirin 5.0 (±2.2 SD) mL/day<br>Fenoprofen 2.5 (±1.2) mL/day<br>Placebo 0.8 (±0.6) mL/day	Individual patient data available<br>8/16 on aspirin had daily blood loss >5 mL/day
Welch et al. Gastroenterol1978 74: 459-463<br>R = 1<br>DB = 0<br>W = 1<br>QS = 2	Cr51-labelled RBC to measure faecal erythrocyte excretion in controlled conditions over two 28-day crossover periods<br>Patients (RA, OA)(n=22) taking at least 2,600 mg aspirin daily<br>Aspirin + placebo<br>Aspirin + cimetidine	Median daily blood loss over last 4 days of treatment:<br>Aspirin + placebo 4.1 (±0.7 SEM) mL/day<br>Aspirin + cimetidine 2.2 (±0,3) mL/day	4/22 patients on aspirin + placebo had mean daily faecal blood loss >5 mL/day, and 1/22 >10 mL/day<br><br>Note randomisation to cimetidine, not aspirin<br><br>Individual patient data available from graph

DeSchepper et al. Clin Pharmacol Ther 1978 23:669-676<br>R = 1<br>DB = 2<br>W = 1<br>QS = 4	Cr51-labelled RBC to measure faecal erythrocyte excretion in controlled conditions over two 7-day periods with 7-day washout (Note parallel study, phase 1 data used)<br>Healthy men aged 20-31 years<br>Aspirin 3000 mg daily (n=5)<br>Diflusinal 500 mg daily (n=5)	Baseline faecal blood loss average 0.32 mL/day<br>Mean daily blood loss over last five days of treatment:<br>Aspirin 6.9 mL/day<br>Diflusinal 0.32 mL/day	1/5 on aspirin had average dauily blood loss >10 mL/day<br><br>Individual patient data available
Baltes. J Clin Pharm 1977 17: 120-124<br>R = 1<br>DB = 1<br>W = 0<br>QS = 2	Cr51-labelled RBC to measure faecal erythrocyte excretion in controlled conditions over two 7-day crossover periods<br>Healthy male volunteers age 21-50 years (average 25 years) (n=19)<br>Aspirin 1,800 mg daily<br>Nefopam 180 mg daily	Baseline faecal blood loss 0.5 mL/day<br>Median daily blood loss over last 3 days of treatment:<br>Aspirin 1.6 (±1.2 SD) mL/day<br>Nefopam 0.6 (±0.4) mL/day	No individual had ≥5 mL/day blood loss on treatment<br><br>Individual patient data available
Vakil et al. Curr Med Res Op 1977 5: 32-37<br>R = 1<br>DB = 2<br>W = 1<br>QS = 4	Cr51-labelled RBC to measure faecal erythrocyte excretion in controlled conditions over 7 days<br>Healthy male volunteers aged 20-40 years<br>Flurbiprofen 150 mg daily (n=6)<br>Phenylbutazone 600 mg daily (n=6)<br>Aspirin 2,100 mg daily (n=6)<br>Placebo (n=6)	Baseline faecal blood loss under 1 mL/day (average 0.8 mL/day)<br>Average daily blood loss over last 4 days of treatment:<br>Flurbiprofen 2.5 (±2.6 SD) mL/day<br>Phenylbutazone 1.3 (±1.5) mL/day<br>Aspirin 3.2 (±1.4) mL/day<br>Placebo 0.7 (±0.4) mL/day	
Loebl et al. JAMA 1977 237: 976-981<br>R = 2<br>DB = 2<br>W = 1<br>QS = 5	Cr51-labelled RBC to measure faecal erythrocyte excretion in controlled conditions over two 7-day crossover periods separated by two weeks using paracetamol<br>Patients with RA age 47-66 years, 6 male 8 female (n=14)<br>Aspirin 4,000 mg daily<br>Fenoprofen 2,400 mg daily	Baseline faecal blood loss not reported<br>Average daily blood loss over last 4 days of treatment:<br>Aspirin 5.0 mL/day<br>Fenoprofen 2.2 mL/day<br>Paracetamol washout 0.8 mL/day	5/11 patients had daily faecal blood loss >5 mL/day on aspirin, and 2/11 >10 mL/day<br>1/12 patients on fenoprofen had blood loss >5 mL/day<br><br>Individual patient data available

Mintz & Fraga. Curr Med Res Op 1976 4: 89-93<br>R = 1<br>DB = 0<br>W = 1<br>QS = 2	Cr51-labelled RBC to measure faecal erythrocyte excretion in controlled conditions over 14 days<br>Patients with RA, 2 male 18 female<br>Azapropazone 600 mg daily (n=10)<br>Azapropazone 1,200 mg daily (n=10)	Average daily blood loss over last 7 days of treatment:<br>Azapropazone 600 mg 1.5 mL/day<br>Azapropazone 1,200 mg 0.5 mL/day	1/10 on azapropazone 600 mg had mean faecal blood loss >5 mL/day<br><br>Individual patient data available
Cohen. Clin Pharm Ther 1976 20: 238-240<br>R = 1<br>DB = 2<br>W = 1<br>QS = 4	Cr51-labelled RBC to measure faecal erythrocyte excretion in controlled conditions over 14 days<br>Men aged 21-45 years (mean 28 years)<br>Sulindac 240 mg daily (n=10)<br>Sulindac 400 mg daily (n=10)<br>Aspirin 4,800 mg daily (n=10)<br>Placebo (n=10)	Baseline faecal blood loss 0.4 mL/day<br>Daily blood loss over last day of treatment:<br>Sulindac 240 mg 0.8 mL/day<br>Sulindac 400 mg daily 0.7 mL/day<br>Aspirin 4,800 mg daily 3.7 mL/day<br>Placebo 0.9 mL/day	1/10 on aspirin had daily faecal blood loss of >5 mL/day<br><br>No dispersion given
Leonards & Levy. Clin Pharm Ther 1973 14: 62-66<br>R = 1<br>DB = 0<br>W = 1<br>QS = 2	Cr51-labelled RBC to measure faecal erythrocyte excretion in controlled conditions over two 7-day periods with washout<br>Healthy volunteers, 5 male 8 female, mean age 23 years<br>Aspirin 3,900 mg daily<br>Sodium salicylate 3,900 mg daily	Baseline faecal blood loss 0.7 mL/day<br>Average daily blood loss over 7 days of treatment:<br>Aspirin 6.3 mL/day<br>Sodium salicylate 1.9 mL/day	5/13 had daily faecal blood loss >5 mL/day on aspirin, and 2/13 >10 mL/day<br><br>Individual patient data available
Leonards & Levy. Arch Intern Med 1972 129: 457-460<br>R = 1<br>DB = 0<br>W = 1<br>QS = 2	Cr51-labelled RBC to measure faecal erythrocyte excretion in controlled conditions over four 7-day periods with washout<br>Healthy volunteers, 7 male 8 female, mean age 26 years<br>Four different types of aspirin formulation, each at 2,600 mg daily	Baseline faecal blood loss 0.4 mL/day<br>Average daily blood loss over 7 days of treatment:<br>A 1.1 (±0.5 SD) mL/day<br>B 1.5 (±0.6) mL/day<br>C 1.1 (±0.6) mL/day<br>D 2.7 (±0.9) mL/day	1/15 had daily faecal blood loss >5 mL/day on aspirin<br>Only preparation D was commercially available<br><br>Individual patient data available

Leonards. J Lab Clin Med 1969 74: 911-914<br>R = 1<br>DB = 2<br>W = 1<br>QS = 4	Cr51-labelled RBC to measure faecal erythrocyte excretion in controlled conditions over four 7-day periods with washout<br>Healthy volunteers, 11 male 1 female, age 19-33 years<br>Aspirin 3,400 mg daily<br>Placebo<br>(plus two experimental combinations)	Baseline faecal blood loss not measured<br>Average daily blood loss over 7 days of treatment:<br>Aspirin 4.8 (±5.5 SD) mL/day<br>Placebo 0.6 (±0.4) mL/day	2/12 had daily faecal blood loss >10 mL/day on aspirin<br><br>Individual patient data available
Rider et al. Clin Ther Res 1965 7: 633-638<br>R = 1<br>DB = 2<br>W = 1<br>QS = 4	Cr51-labelled RBC to measure faecal erythrocyte excretion in controlled conditions over 14 days<br>Normal volunteers in good health<br>Aspirin 1,800 mg daily (n=8)<br>Namoxytrate 1,800 mg daily (n=8)	Baseline faecal blood loss 0.4 mL/day<br>Daily blood loss over last 2 days of treatment:<br>Aspirin 4.0 (±1.0 SEM) mL/day<br>Namoxytrate 5.8 (±2.0) mL/day	3/8 on aspirin had daily faecal blood loss of >5 mL/day<br>4/8 on namoxytrate had daily faecal blood loss of >5 mL/day<br><br>Individual patient data available

Abbreviations: R = randomised; SB = double blind; W = withdrawals and dropouts; QS = quality score; SD = standard deviations; OA = osteoarthritis; RA = rheumatoid artyhritis; AS = ankylosing spondilitis
